# AI-driven assessment of over-scanning in chest CT: A systematic review and meta-analysis

**DOI:** 10.1016/j.ejro.2025.100674

**Published:** 2025-07-30

**Authors:** Mo’men Bani-Ahmad, Andrew England, Laura McLaughlin, Marwan Alshipli, Kholoud Alzyoud, Yasser H. Hadi, Mark McEntee

**Affiliations:** a The Discipline of Medical Imaging and Radiation Therapy, University College Cork, College Road, Cork T12 K8AF, Ireland; b Syddansk Universitet - University of Southern Denmark Faculty of Health Sciences, Denmark; c University of Sydney, Faculty of Medicine, Australia; d Faculty of Applied Medical Sciences, Department of Medical Imaging, The Hashemite University, Zarqa, Jordan; e Department of Medical Imaging and Radiography, Aqaba University of Technology, Aqaba, Jordan; f Faculty of Applied Medical Sciences, Al Al-Bayt University, Mafraq, Jordan

**Keywords:** Quality control, Anatomic coverage, Scan range, Deep learning, Machine learning

## Abstract

**Introduction:**

Scan range is crucial for CT acquisitions. However, irrelevant over-scanning in CT is common and contributes to a significant radiation dose. This review explores the role of artificial intelligence (AI) in addressing manual over-scanning in chest CT imaging.

**Methods:**

A systematic search of peer-reviewed publications was conducted between December 2015 and March 2025 in Embase, Scopus, Ovid, EBSCOhost, and PubMed. Two reviewers and an academic lecturer independently reviewed the articles to ensure adherence to inclusion criteria. The quality of the included studies was assessed using CLAIM and QUADAS-2 tools. Summary estimates on over-scanning at the upper and lower boundaries of the scan range in chest CT were derived using meta-analysis.

**Results:**

Five studies employed AI algorithms to assess manual over-scanning in chest CT using either 2D topograms or 3D axial images at low and standard doses. These models accurately determine the extent of over-scanning, demonstrating strong agreement with radiologist evaluations. All included studies revealed significant variation in over-scanning at the superior (13.5 mm) and inferior (30.2 mm) boundaries of the scan range (p < 0.001), with approximately two-thirds of the total over-scanning (43.2 mm) occurring at the inferior level (abdomen).

**Conclusions:**

Integrating AI tools into the over-scanning evaluation process may optimise chest CT imaging protocols and enhance patient safety by reducing over-scanning and radiation dose through real-time monitoring and retrospective analysis.

## Highlights

• AI models accurately assess over-scanning in chest CT, aligning with radiologist evaluations.
• AI tools may optimise chest CT scanning, reducing over-scanning and radiation dose.
• AI-based real-time and retrospective analysis of over-scanning instances may enhance patient safety.

## Graphical Abstract

**Figure:**
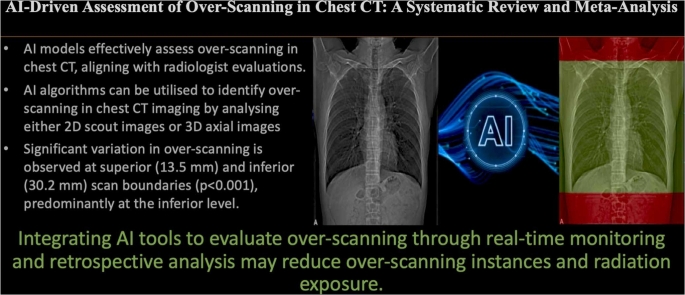

## 1 Introduction

Chest computed tomography (CT) is a cornerstone in the diagnosis of thoracic diseases, offering high-resolution imaging of the lungs, mediastinum, pleura, and chest wall [1]. The demand for chest CT has grown significantly, particularly during (116 %) and following (71 %) the COVID-19 pandemic [2]. However, a key limitation of CT is its relatively high radiation dose compared to other imaging modalities, such as chest radiography [3]. Exposure to ionising radiation is associated with an elevated risk of carcinogenesis, especially in younger patients and those undergoing repeated imaging [4]. A recent study estimated that approximately 2.5 million patients received a cumulative effective dose of ≥ 100 mSv from CT examinations over a five-year period [5]. If current trends persist, CT scans performed in 2023 alone could contribute to an estimated 103,000 future cancer cases, potentially accounting for 5 % of all new annual cancer diagnoses [6]. These figures underscore the urgent need to reduce radiation exposure in clinical practice.

Although advances in scanner technology and image reconstruction have contributed to dose reduction, substantial variation in scanning parameters and radiation doses remains [7] A key contributor to unnecessary radiation is over-scanning, which occurs when the scan range extends beyond the clinically relevant anatomical region [8]. In the context of chest CT, over-scanning often includes areas such as the lower neck or upper abdomen, which lie outside the clinically relevant thoracic region and are not required for diagnostic purposes [9].

Unlike over-beaming—a technical limitation inherent to helical CT acquisition—over-scanning is avoidable and can be mitigated through precise scan range determination [10]. can range selection is typically performed manually by radiographers using anatomical landmarks on a 2D topogram [11], [12]. However, this process is susceptible to variability due to differences in radiographer experience, workload, and patient characteristics [12], [13]. As a result, over-scanning remains a common issue in chest CT, with multiple studies reporting high prevalence rates and highlighting the need for improved protocols to minimise unnecessary radiation exposure [14], [15], [16], [17], [18].

Artificial intelligence (AI) offers a promising solution for monitoring scan ranges in chest CT scans [19]. AI can analyse large datasets of CT scans to identify patterns and instances of over-scanning, providing valuable insights into its frequency and contributing factors [20]. Implementing AI as an audit tool in chest CT scans can enhance quality control measures by offering retrospective analysis and detailed quality control reports [21], [22]. Furthermore, AI has the potential to support real-time monitoring, offering immediate feedback to technologists to rectify deviations from optimal scanning protocols [23].

This systematic review aims to evaluate the potential of AI as a monitoring tool to ensure appropriate anatomical coverage and reduce over-scanning in chest CT, thereby minimising radiation exposure and enhancing patient safety.

## 2 Materials and methods

Reporting of this systematic review followed the Preferred Reporting Items for Systematic review and Meta-Analysis Protocols (PRISMA) guidelines [24]. The PRISMA checklist was rigorously adhered to throughout both the design and reporting stages to maintain methodological integrity (Supplemental Table S1).

### 2.1 Search strategy

Two independent reviewers (M.B.A and M.A) conducted a comprehensive literature search to identify studies using AI methodologies for evaluating over-scanning in chest CT examinations. Systematic searches were performed across the Embase, Scopus, Ovid, EBSCOhost, and PubMed databases from December 2015 to March 2025. The search strategy included the following keywords: *(scan range OR scan length OR scan coverage OR acquisition length OR acquisition range OR overscanning OR over-scanning OR over-scan OR scan area OR scan volume OR scan extent OR scan boundary OR scan limit) AND (computed tomography OR CT) AND (chest OR thorax OR lung) AND (artificial intelligence OR machine learning OR deep learning OR neural networks).* Detailed results of the search are provided in Supplemental Table S2. The inclusion criteria were restricted to studies on chest CT that employed AI techniques to evaluate over-scanning. Excluded from this review were prior reviews, meta-analyses, conference abstracts, editorials, letters, surveys, and studies utilising non-CT imaging modalities or non-AI methodologies. No language limitations were applied during the initial search. However, only English-language articles were included in the final analysis, as no relevant studies in other languages were identified.

## 3 Study selection and data collection

The identified studies were imported into the Covidence platform (Melbourne, Victoria, Australia) in RIS text format, and duplicates were removed. Two reviewers (M.B.A & M.A) independently and blindly screened the titles and abstracts of all studies. Subsequently, full-text articles were uploaded for further screening, with reasons for exclusions during the full-text review being documented. Any disagreements or uncertainties regarding the inclusion or exclusion of an article were resolved by consensus through discussion with a third researcher (K.A) to determine the final eligibility of the studies.

### 3.1 Data extraction and quality assessment

M.B.A. and M.A. independently and in duplicate extracted data from eligible articles using structured forms. These forms included details on study design, patient demographics, clinical settings, network models, training and validation processes, quantitative measurement tools, and instances of over-scanning. The methodological quality of each included study was rigorously evaluated using the Checklist for Artificial Intelligence in Medical Imaging (CLAIM) and the Quality Assessment of Diagnostic Accuracy Studies-2 (QUADAS-2) tools [25], [26]. Two authors (M.B.A & K.A) independently conducted the quality assessment. In case of differing interpretations, senior researchers (A.E) were consulted to resolve these discrepancies. This dual-layered approach ensured a comprehensive and unbiased evaluation of methodological quality. The CLAIM checklist assessed AI-specific methodological rigor, while QUADAS-2 addressed risk of bias in diagnostic accuracy. Their combined use provided complementary insights aligned with the dual focus of the included studies.

### 3.2 Statistical analysis

A meta-analysis was conducted using Jamovi 2.4.13 software (Jamovi, Greensboro, NC), employing random-effects models to evaluate the efficiency of different AI models in determining over-scanning at the upper and lower boundaries of the scan range of chest CT. Statistical heterogeneity was assessed using standardised mean differences, tau², Q-test, and I² statistics. Prediction intervals, studentised residuals, and Cook's distances were utilised to identify outliers or influential studies. In addition to the meta-analysis, a paired samples *t*-test was performed to compare the mean over-scanning at the superior and inferior boundaries of the chest CT scan range. Each of the four included studies reported both measurements using consistent anatomical landmarks (pulmonary apex and costophrenic angle), allowing for study-level pairing. Normality of the paired differences was assessed using the Shapiro-Wilk test and confirmed via Q-Q plot inspection.

## 4 Results

### 4.1 Study selection

**Fig. 1:**
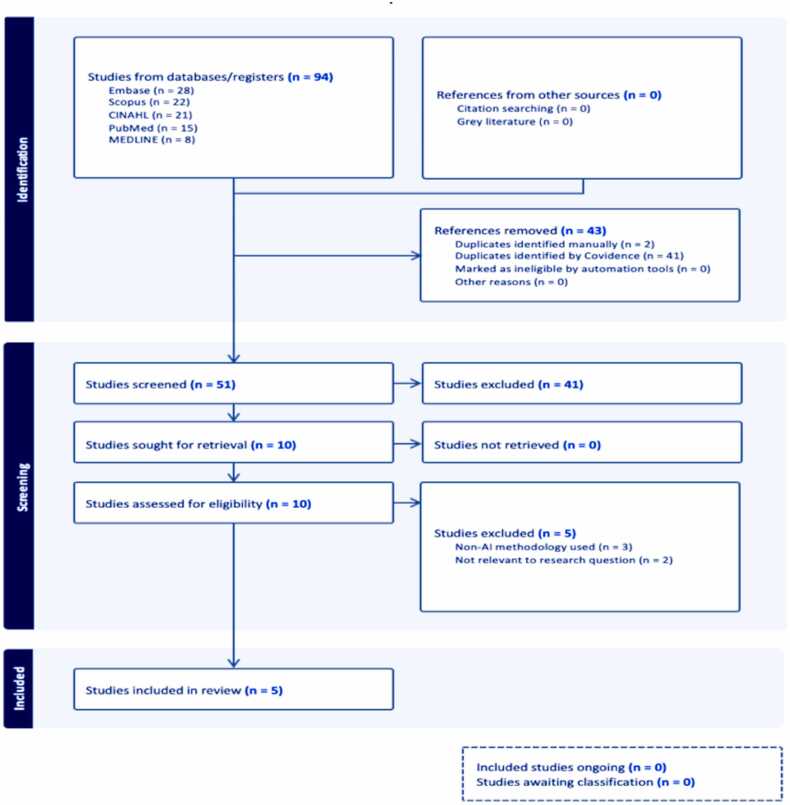
**PRISMA flowchart.** This flowchart illustrates the study selection process following PRISMA guidelines. It details the number of records identified, screened, excluded, and included in the final systematic review, along with reasons for exclusion at each stage.

The initial search across various databases yielded 94 studies, including 28 from EMBASE, 22 from Scopus, 21 from CINAHL, 15 from PubMed and 8 from MEDLINE, as illustrated in the PRISMA flowchart (Fig. 1**)**. After removing 43 duplicates, we used Covidence software to screen the remaining 51 articles. By screening titles and abstracts, we excluded 41 articles due to their lack of relevance. Further examination of the remaining 10 full-text articles led to the exclusion of five articles. The reasons for exclusion were articles irrelevant to the systematic search question or not using AI methodologies. Finally, this rigorous selection process resulted in five studies [19], [20], [21], [22], [23] being included in our systematic review.

### 4.2 Study characteristics

Table 1 presents a comprehensive summary of five studies conducted between 2019 and 2022, involving 22,964 patients. These studies exhibited substantial differences in patient enrolment, with the number of patients ranging from 260 [13] to 20,820 [20]. A retrospective design was used in these studies, which focused on screening and diagnosis. Four studies employed distinct convolutional neural network (CNN) architectures (including 2D ResNet and U-Net) as evaluation tools for over-scanning, whereas one study [22] did not specify the model utilised. A common methodological pattern involved dividing datasets into separate training and testing sets; however, only Kaviani et al. [22] employed cross-validation techniques. These studies consistently employed manual annotation and expert-defined ground-truth methods, highlighting a prevalent reliance on traditional, expert-driven analytical approaches. The sizes of datasets used for training varied significantly, ranging from 50 [23] to 13,280 [20]. Data augmentation, which enhances training data diversity through various techniques such as rotation, scaling, translation, zooming and flipping, is used to enhance model generalisability by exposing the models to a broader range of data scenarios [19], [21], [23]. The included studies employed various approaches to identify over-scanning in CT scans: three used segmentation [20], [21], [23], one used classification to categorise images into cervical, lung, and abdomen classes [19], and one used a combination of classification and segmentation [22]. Segmentation delineates anatomical structures and regions of interest within the CT images, while the combination approach integrates both tasks to enhance the identification process. The choice of loss functions, including cross-entropy loss, dice loss, or a combination of both, varied across studies, indicating the use of diverse strategies for optimising segmentation accuracy. Performance metrics such as dice similarity coefficient (DSC), intersection over union (IOU), area under the curve (AUC), accuracy, sensitivity, and specificity were calculated to assess model performance. Three studies [19], [22], [23] used actual (axial) CT images to quantify the extent of over-scanning, whereas two studies [20], [21] employed scout (2D) images for this purpose. The ideal scan length was determined in four studies [19], [20], [21], [22] as the distance from the uppermost pulmonary apex (superior boundary) to the lowermost costophrenic angle (inferior boundary). In contrast, Kim et al. [23] identified the distance from the upper end of the thyroid cartilage to the lower end of the kidney as standard reference points.

**Table 1:** Study characteristics and AI algorithms.

**Author,** **Year, Country**	**Study Design**	**Clinical setting**	**Patients**	**CT images**	**Algorithms**	**Reference**	**Validation**	**Task type**
Colevrary et al. [19] 2019 France	Retrospective	Screening	1000	3D (Axial)	CNN	Manual	Random split sample validation	Classification
Salimi et al. [20] 2021 Iran	Retrospective	Diagnostic	20,820	2D (AP and lateral scout views)	2D ResNet	Automated	Random split sample validation	Segmentation
Huo et al. [21] 2019 USA	Retrospective	Screening	770	2D (AP scout)	U-Net	Manual	Random split sample validation	Segmentation
Kaviani et al. [22] 2022 USA	Retrospective	Diagnostic	428	3D (Axial)	NR	Manual	Five-fold cross-validation	Classification and segmentation
Kim et al. [23] 2022 Republic of korea	Retrospective	Screening	260	3D (Axial)	U-Net	Manual	Random split sample validation	Segmentation

This table summarizes the key characteristics of the included studies, including author, year, country, study design, clinical setting, number of patients and CT images, AI algorithms used, reference methods, validation strategies, and task types.

### 4.3 Quality assessments

**Fig. 2:**
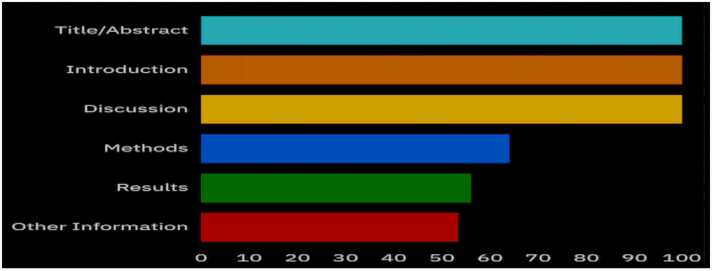
**Studies’ compliance with CLAIM criteria.** This flowchart illustrates the study selection process following PRISMA guidelines. It details the number of records identified, screened, excluded, and included in the final systematic review, along with reasons for exclusion at each stage.

Table 2 and Fig. 2 present the results of a comprehensive assessment of the five studies performed using the CLAIM criteria. These studies reported a mean CLAIM score of 29.6, approximately 67.27 %, ranging from 26 to 34 out of a possible total score of 44. The average scores for CLAIM subsections further revealed the quality of these five studies; the breakdown is as follows: title/abstract, 100 %; introduction, 100 %; methods, 19.2/30 (64 %); results, 2.8/5 (56 %); discussion, 2/2 (100 %); and other information, 1.6/3 (53.3 %). These findings highlight the strengths of these five studies and potential areas for improvement in chest CT by applying AI methodologies to monitor over-scanning in scan range. The QUADAS-2 results show that most of the included studies had a low risk of bias in patient selection, index tests, and reference standards, with all studies consistently low in flow and timing (Table 3). However, the study by Kim et al. [23] exhibited a high risk of bias due to its reference standard (the upper end of the thyroid cartilage to the lower end of the kidney), which does not logically align with the typical anatomical boundaries for chest CT scans (lung apices to costodiaphragmatic recess). Despite this high risk of bias, the study was included in the review due to its relevance to AI-based over-scanning evaluation and because the exclusion criteria applied only to studies with a high risk of bias in three or more domains.

**Table 2:** Summary of the results of the CLAIM quality assessment.

**Study**	**Title/Abstract**	**Introduction**	**Methods**							**Results**		**Discussion**	**Other Information**	**Total Score**
			**Study design**	**Data**	**Reference Standard**	**Data preparation**	**Model**	**Training**	**Evaluation**	**Data**	**Model performance**			
	**(2)**	**(2)**	**(2)**	**(7)**	**(5)**	**(3)**	**(3)**	**(3)**	**(7)**	**(2)**	**(3)**	**(2)**	**(3)**	**(44)**
Colevray et al. [19]	2	2	2	3	3	2	2	2	4	1	2	2	1	28
Salimi et al. [20]	2	2	2	5	4	2	3	2	5	1	2	2	2	34
Huo et al. [21]	2	2	2	4	4	2	2	2	4	1	2	2	2	31
Kaviani et al. [22]	2	2	2	4	3	2	2	1	3	1	1	2	1	26
Kim et al. [23]	2	2	2	4	3	2	2	2	3	1	2	2	2	29

This table presents the CLAIM (Checklist for Artificial Intelligence in Medical Imaging) quality assessment scores for each study across multiple domains, including study design, data handling, model development, training, evaluation, and reporting. Total scores are also provided.

**Table 3:** Summary of the results of the QUADAS-2 risk of bias assessment [19], [20], [21], [22], [23].

This table outlines the risk of bias and applicability concerns for each study based on the QUADAS-2 tool, covering domains such as patient selection, index test, reference standard, and flow and timing.

### 4.4 Over-scanning

**Fig. 3:**
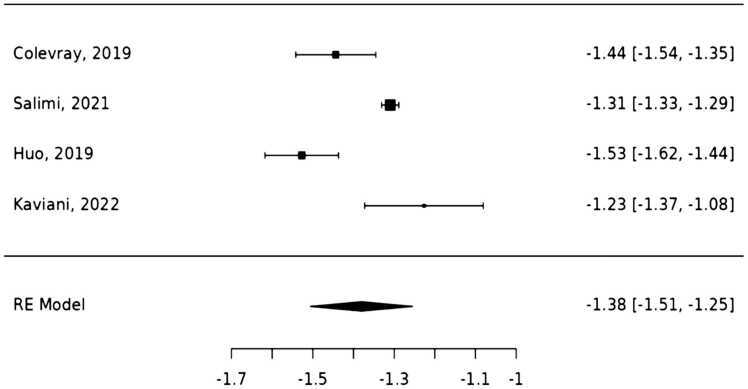
**Forest plot of effect sizes from studies comparing manual over-scanning at the upper and lower boundaries using various AI models, accompanied by a random-effects model summary.** This plot illustrates a meta-analysis of over-scanning in scan ranges determined by AI methods. Each row represents a distinct study, with squares indicating the mean effect size and horizontal lines showing the 95 % confidence intervals. The pooled effect size suggests substantial over-scanning from the inferior boundaries of scan ranges compared to the superior boundaries.

This review incorporated findings from five research studies on the evaluation of over-scanning in chest CT examinations using AI methods (Table 4). One study [23] was excluded from the analysis due to the absence of data on over-scanning at the upper and lower boundaries of the chest CT scan range. The meta-analysis included four studies (Fig. 3), with observed standardised mean differences ranging from − 1.53 to − 1.23, all negative. The average standardised mean difference was − 1.38 (95 % CI: − 1.51 to − 1.25), significantly different from zero (z = -21.47, p < 0.001). Despite substantial heterogeneity (Q (3) = 28.41, p < 0.001, tau² = 0.01, I² = 90.1 %), the true outcomes were generally consistent, as indicated by the 95 % prediction interval (-1.65 to − 1.12). No outliers or overly influential studies were identified based on studentised residuals and Cook's distances. Additionally, there was no evidence of funnel plot asymmetry (p = 1.00 and p = 0.90 respectively). Subgroup or sensitivity analyses were not conducted due to the limited number of included studies, which constrained further exploration of heterogeneity.

**Table 4:** Summary of mean over-scanning, effective over-scanning dose, and model performance.

Study	Mean over-scanning (mm)			Mean effective over-scanning dose (mSv)	Model performance
	Superior	Inferior	Overall		
Colevray et al. [19]	23.3 ± 8.7	51.2 ± 25.9	74.5 ± 27.3	NR	Accuracy 0.98 Kappa _INTER-RATER_ = 0.98 Kappa _validation_= 0.96
Salimi et al. [20]	20.2 ± 7.7	31.2 ± 9.0	51.4 ± 11.8	2.6	DSC 0.96
Huo et al. [21]	17 ± 3.1	41 ± 20.1	58.5 ± 20.3	1.24	DSC 0.98 IOU 0.95
Kaviani et al. [22]	19.8 ± 7.9	40.5 ± 22.5	60.3 ± 23.9	NR	Sensitivity 0.98 Specificity 0.98 Accuracy 0.98 AUC 0.99
Kim et al. [23]	NR	NR	16.3 ± 1.06	0.035	Accuracy 0.96 Sensitivity 0.95 Specificity 0.96 AUC 0.98 Kappa values 0.91 DSC 0.86

* NR; not reported, DSC; dice similarity coefficient, IOU; intersection over Union, AUC; area under the curve.

*Kim et al. [23] was included in this table for completeness but excluded from the meta-analysis due to incompatible outcome data.

This table reports the mean over-scanning (superior, inferior, and overall), effective over-scanning doses, and performance metrics of the AI models used in each study. Performance metrics include accuracy, sensitivity, specificity, AUC, DSC, IOU, and kappa values.

The analysis demonstrates that AI consistently identified less over-scanning at the upper boundaries of the scan range compared to the lower boundaries. This significant difference in over-scanning measurements between the superior and inferior boundaries of the chest CT scan indicates that over-scanning is more pronounced at the inferior (abdominal) boundary. The consistency of these findings across multiple studies, along with the significant statistical results, supports the reliability of AI in evaluating manual over-scanning in chest CT scans. Furthermore, the funnel plot analysis of the four studies and Egger’s regression test yielded a p-value of 0.9, indicating no significant publication bias, thus supporting the robustness of the results.

**Fig. 4:**
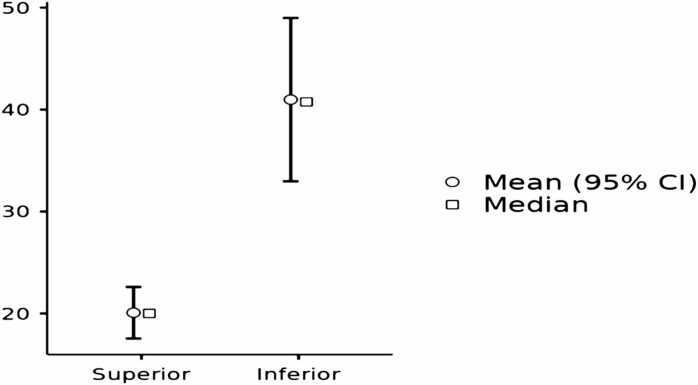
**Comparison of over-scanning extent at superior and inferior scan range boundaries.** This figure compares the extent of over-scanning at the superior and inferior boundaries of the chest CT scan range. It shows that over-scanning from the inferior boundary is significantly greater than from the superior boundary.

**Fig. 5:**
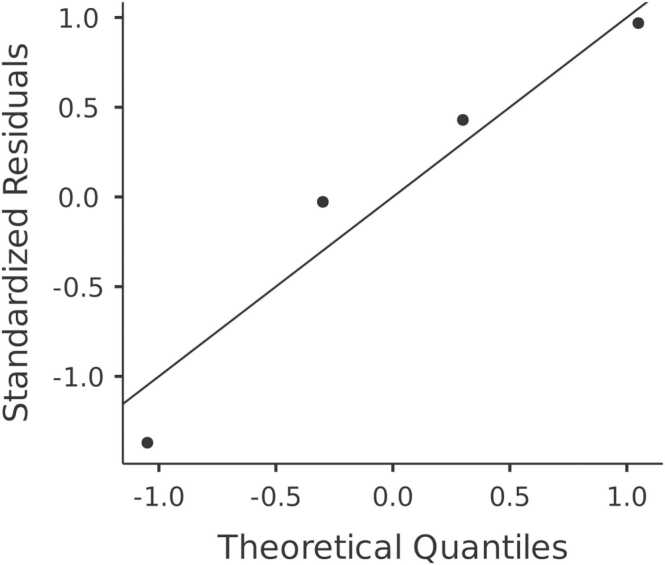
**Q-Q plot of the differences in over-scanning between superior and inferior boundaries.** This Q-Q plot assesses the normality of the differences in over-scanning measurements between the superior and inferior scan boundaries. The plot supports the assumption of normal distribution, validating the use of parametric statistical tests.

The paired samples *t*-test revealed that the extent of over-scanning was significantly greater at the inferior boundary compared to the superior boundary of the chest CT scan range. The mean difference was − 20.9, with a t-statistic of − 5.78 and a p-value of 0.010, indicating statistical significance. Descriptive statistics (Fig. 4), showed a mean over-scanning of 20.1 mm at the superior boundary and 41.0 mm at the inferior boundary. The Shapiro-Wilk test confirmed that the differences did not significantly deviate from normality (W = 0.944, p = 0.681), which was further supported by visual inspection of the Q-Q plot (Fig. 5).

The evaluation of AI models for over-scanning in chest CT across multiple studies demonstrated consistently high performance. The models showed excellent accuracy, sensitivity, specificity, and overall agreement, with notable AUC, Kappa values, and DSC metrics. Three studies [19], [20], [21] provided mean differences between AI models and radiologists of 5.6 mm, 3.5 mm, and 10.55 mm, respectively. These differences are relatively small despite variability, indicating AI models are generally close to radiologists' measurements. Although direct statistical comparison data is unavailable, the mean differences, along with evaluation metrics and the meta-analysis results, indicate that AI models are reliable tools for assessing over-scanning in chest CT, providing robust and consistent performance across different studies.

Three studies [20], [21], [23] reported radiation doses associated with over-scanning: Salimi et al. [20] (2.6 mSv), Huo et al. [21] (1.24 mSv) and Kim et al. [23] (0.03 ± 0.04 mSv). The lower dose observed in Kim's study [23] can be attributed to the reference standard for ideal scan coverage, from the upper end of the thyroid cartilage to the lower end of the kidney. In contrast, Huo and Salimi's studies [20], [21] used the lung apex and costophrenic angle, which are more accurate concerning lung anatomy. Additionally, Kim's study [23] had a lower mean over-scanning length (16 mm) than other studies, with Colevray et al. [19] reporting the highest (74 mm).

## 5 Discussion

This is the first systematic review, to the best of our knowledge, examining the role of AI in assessing over-scanning in chest CT imaging. Our findings highlight the ability of various AI algorithms to determine manual over-scanning with high accuracy, optimising CT imaging procedures.

The analysis indicated that over-scanning at the inferior boundaries was significantly higher than at the superior boundaries (p < 0.01), with approximately two-thirds of over-scanning occurring at the at the inferior boundary of the scan range (abdominal region). This can be attributed to several factors, including the high degree of heterogeneity in imaging the lower part of the lung, which complicates the delineation of anatomical structures. This variability in tissue density and composition makes it challenging to determine the scan range accurately [19]. Moreover, patient-related factors such as breathing and body size, along with the radiographer's experience and the tendency to extend the scan range to avoid missing critical anatomical details, also play a critical role in increasing over-scanning inferiorly [20]. While this practice ensures diagnostic accuracy, it contributes to increased radiation exposure to patients [4], [14], [18]

These findings are consistent with previous literature. For instance, Zanca et al. [14] conducted a study involving 167 consecutive patients undergoing chest and/or abdominal examinations and observed that up to 80 % of thoraco-abdominal CT scans experienced over-scanning. The average additional scanning length was 1.8 cm superiorly and 2.9 cm inferiorly, totalling an extra imaging length of 4.7 cm. Similarly, Yar et al. [15] reported that out of 2032 CT examinations, which included thorax, abdominopelvic, thoraco-abdominopelvic, pulmonary CT angiography, and urinary stone scans, 66 % experienced over-scanning, particularly in the inferior direction (48.5 %). The highest rate of over-scanning was observed in thorax scans, with 89.2 % of cases exhibiting this issue. Schwartz et al. [16] found that over-scanning occurred in up to 60 % of chest CT scans, with a higher incidence of caudal over-scanning when only an AP scout was used (33.7 %) compared to using both AP and lateral scouts (15.3 %).

The differences in radiation doses reported by three studies [20], [21], [23] underscore the variability in over-scanning practices and their impact on patient exposure. Salimi et al. [20] and Huo et al. [21] reported relatively high doses of 2.6 mSv and 1.24 mSv, respectively, using lung boundaries as a ground truth, indicating more extensive over-scanning. Furthermore, Salimi's findings indicate that over-scanning at the lower boundary could contribute to a radiation dose of 1.9 ± 1.1 mSv, approximately 2.5 times higher than that from the upper boundary (0.75 ± 0.6 mSv). In contrast, Kim et al. [23] reported an excessive dose of 0.03 mSv, despite the ideal scan length in their study exceeding the anatomical boundaries of the chest. These variations primarily reflect differences in how over-scanning is defined and measured. However, they may also be influenced by contextual factors such as institutional scan protocols or regional imaging practices, which can affect how scan boundaries are set and interpreted. Recognising these influences reinforces the need for standardised definitions and evaluation methods when assessing over-scanning in chest CT.

The potential role of AI in determining over-scanning is significant, as it can directly impact patient dose by identifying and reducing unnecessary scan lengths. AI models can provide real-time monitoring and retrospective analysis, thereby improving the efficacy and radiation safety of chest CT imaging. The included studies employed 2D localiser images or 3D axial chest CT scans to train AI models for detecting over-scanning. Utilising 2D localisers offers real-time monitoring benefits, as the topogram defines the anatomy to be scanned before imaging, enabling proactive scan range recommendations, which reduce workload and inter-operator variability [21]. In contrast, axial CT scans provide a retrospective evaluation of over-scan length and associated excessive doses, aiding in preventing over-scanning through detailed quality control reports and comprehensive retrospective analysis [23]. This dual approach fosters improvements in imaging practices, both in real-time and through long-term quality control measures, resulting in enhanced patient safety by minimizing unnecessary radiation exposure and ensuring adherence to standardised protocols.

To further illustrate the accuracy and efficiency of AI models in this context, Colevray et al. [19] found a strong agreement (kappa = 0.98) between the CNN and radiologists' evaluations. Their analysis of 1000 lung CT examinations revealed a 22.6 % degree of over-scanning, with a significantly higher incidence of systematic over-scanning in the abdomen, observed at 87.5 %. Additionally, the CNN required a mean computation time of 23.04 s per examination to determine over-scanning, highlighting its efficiency and suitability for clinical settings. In the studies by Kim et al. [23] and Kaviani et al. [22], evaluation metrics indicated that age, sex, data source, CT vendor, and slice thickness did not statistically influence the performance of DL models or the algorithm's decisions (p > 0.1). These results suggest that the DL models are robust and generalisable across diverse patient demographics and imaging conditions, highlighting their potential for widespread clinical application.

The extent of over-scanning in chest CT is influenced by several factors, as evidenced by the studies conducted by Huo et al. [21] and Salimi et al. [20]. According to Huo's study [21] the technologist's experience and patient weight significantly affect over-scanning, whereas acquisition date, time, and patient age do not exhibit a significant impact. Conversely, Salimi's study [20], observed that over-scanning is more frequent in older patients (p > 0.05) and female patients (p < 0.001), with significant positive correlations with patient size and body mass index (BMI). Cohen et al. [17], [18] highlighted a significant correlation between the occurrence of over-scanning and the technologists' workload. These findings underscore the importance of considering both technologist practices and patient characteristics to minimise over-scanning. In this context, AI can play a crucial role in minimising over-scanning by guiding technologists with low experience, and reducing the negative effects on radiographers' focus during high workload periods by providing real-time feedback.

Several factors, including data quality and volume, patient characteristics, data sources, technical variations, and methods for model development and validation, influence the generalisability of the reviewed studies. The studies included 22,964 patients, covering a range of ages, weights, sexes, and countries, enhancing generalisability. Data augmentation techniques, CT scanners, and acquisition parameters were employed, potentially reducing overfitting. External validation was performed in three studies, enhancing the ability to confirm the models' performance on unseen data. Overall, these approaches appear to mitigate overfitting and enhance generalisability, with external validation in most studies ensuring the robustness and applicability of the findings across different settings.

This systematic review has several limitations. The inclusion of only five articles on chest CT may limit the generalisability of the findings. Nonetheless, these studies represent the only available research on the use of AI for evaluating over-scanning in CT imaging. The substantial heterogeneity observed likely reflects differences in AI models, datasets, and anatomical landmarks. While subgroup or sensitivity analyses were not feasible due to data limitations, future studies should explore these sources to enhance interpretability. Additionally, the unavailability of data for direct statistical comparisons between AI methods and standard references (radiologists) in determining over-scanning may affect the accuracy and reliability of the reported results. Despite these limitations, the review highlights the potential role of AI as an effective tool for evaluating over-scanning in chest CT. This review provides a foundation for future research to refine AI models and improve their integration into clinical practice.

## 6 Conclusion

In conclusion, anatomical complexity, patient movement, radiographer experience, and precautionary measures to ensure complete imaging collectively contribute to the observed over-scanning in chest CT, particularly at the lower boundary of the scan range. Integrating AI tools into the over-scanning assessment offers a promising approach for real-time monitoring and retrospective analysis, potentially improving the efficacy and radiation safety of chest CT imaging.

## CRediT authorship contribution statement

**Bani-Ahmad Mo'men:** Writing – original draft, Visualization, Validation, Software, Methodology, Investigation, Formal analysis, Data curation. **Andrew England:** Writing – review & editing, Validation, Supervision, Project administration, Methodology, Formal analysis, Conceptualization. **Laura McLaughlin:** Writing – review & editing, Validation, Supervision. **Marwan Alshipli:** Methodology, Formal analysis, Data curation. **Kholoud Alzyoud:** Methodology, Formal analysis, Data curation. **Yasser H. Hadi:** Software, Methodology, Investigation. **Mark McEntee:** Writing – review & editing, Visualization, Validation, Supervision, Resources, Project administration, Conceptualization.

## Ethics approval and consent to participate

Not applicable.

## Consent for publication

Not applicable.

## Declaration of Generative AI and AI-assisted technologies in the writing process

During the preparation of this work, the author(s) used Microsoft Copilot for Microsoft 365 to assist with improving readability and language. After using Microsoft Copilot, the author(s) reviewed and edited the content as needed and take(s) full responsibility for the content of the published article.

## Funding

This research received no specific grant from any funding agency in the public, commercial, or not-for-profit sectors.

## Declaration of Competing Interest

The authors declare that they have no competing interests.

## Data Availability

The data that support the findings of this study are available from the corresponding author upon reasonable request.
